# Early lineage segregation of the retinal basal glia in the *Drosophila* eye disc

**DOI:** 10.1038/s41598-020-75581-w

**Published:** 2020-10-28

**Authors:** Chia-Kang Tsao, Yu Fen Huang, Y. Henry Sun

**Affiliations:** 1 Department of Life Sciences and Institute of Genome Sciences, National Yang-Ming University, Taipei, Taiwan, ROC; 2 Institute of Molecular Biology, Academia Sinica, Taipei, Taiwan, ROC; 3 Present Address: 64 Marvin Lane, Piscataway, NJ 08854 USA

**Keywords:** Developmental biology, Neurogenesis, Developmental neurogenesis

## Abstract

The retinal basal glia (RBG) is a group of glia that migrates from the optic stalk into the third instar larval eye disc while the photoreceptor cells (PR) are differentiating. The RBGs are grouped into three major classes based on molecular and morphological characteristics: surface glia (SG), wrapping glia (WG) and carpet glia (CG). The SGs migrate and divide. The WGs are postmitotic and wraps PR axons. The CGs have giant nucleus and extensive membrane extension that each covers half of the eye disc. In this study, we used lineage tracing methods to determine the lineage relationships among these glia subtypes and the temporal profile of the lineage decisions for RBG development. We found that the CG lineage segregated from the other RBG very early in the embryonic stage. It has been proposed that the SGs migrate under the CG membrane, which prevented SGs from contacting with the PR axons lying above the CG membrane. Upon passing the front of the CG membrane, which is slightly behind the morphogenetic furrow that marks the front of PR differentiation, the migrating SG contact the nascent PR axon, which in turn release FGF to induce SGs’ differentiation into WG. Interestingly, we found that SGs are equally distributed apical and basal to the CG membrane, so that the apical SGs are not prevented from contacting PR axons by CG membrane. Clonal analysis reveals that the apical and basal RBG are derived from distinct lineages determined before they enter the eye disc. Moreover, the basal SG lack the competence to respond to FGFR signaling, preventing its differentiation into WG. Our findings suggest that this novel glia-to-glia differentiation is both dependent on early lineage decision and on a yet unidentified regulatory mechanism, which can provide spatiotemporal coordination of WG differentiation with the progressive differentiation of photoreceptor neurons.

## Introduction

The nervous system is composed of neurons and glia. Glial cells play essential roles in various aspects of neural development and function in mammals and in *Drosophila*^1–3^. Glia comes in multiple subtypes in fly and mammals^4–6^. How specific glia subtypes are generated and the lineage relationship between glial subtypes is still not well understood.

In *Drosophila*, almost all glia expresses the homeodomain protein Reverse polarity (Repo), which is required to maintain glia fate and to block hemocyte fate^7–9^. Unlike its transiently expressed upstream inducer, Glia cell missing (Gcm)^10^, Repo is stably expressed in glia, thus being widely used as a pan-glia marker.

The *Drosophila* eye imaginal disc is an excellent experimental system to study glial lineages, proliferation, migration and differentiation, as well as glia–neuron and glia–glia interactions. In this system, the retinal basal glia (RBG) migrates into the eye disc during the larval stage, presumably from the brain through the optic stalk (OS)^11^. Their migration initiates after the onset of photoreceptor (PR) differentiation. As the PR progressively differentiate in a posterior-to-anterior direction, the RBGs follow but with a lag behind the anterior front of the differentiating PRs. The RBGs consist of three major cell types: surface glia (SG; also categorized as perineurial glia), wrapping glia (WG; also categorized as ensheathing glia) and carpet glia (CG; also categorized as subperineurial glia)^12–14^. The WG is a special glial subtype only found in the peripheral nervous system (PNS). It wraps axons and functionally resembles the non-myelinating Schwann cells in the mammalian PNS^6^.

There are two CGs per eye disc. They have giant polyploid nucleus and broad membrane extension that each cover half of eye disc. The CGs are defined by their expression of *moody-GAL4*^14^, *C135-GAL4*^15^, *15IIa-GAL4*^16^, the zinc-finger transcription factor Hunchback (Hb), two *hb-GAL4*s (VT038544 and VT038545) and the NCAM Fasciclin 2 (Fas2)^17^. The membrane of the two CGs forms septate junction at the dorsoventral midline (equator)^14^. The CG membrane (visualized by *C135-GAL4* driven *UAS-moesin-GFP*, abbreviated as *C135* > *moesin-GFP*) extends anteriorly but lag a short distance, about 1–2 rows of ommatidia, behind the anterior front of RBGs, which is itself a short distance, about 3–5 rows of ommatidia, behind the developing photoreceptor (PR) clusters (Lee, 2011). The CG membrane forms a carpet that separates the apical and basal surfaces. The PRs are on the apical side of the CG membrane. The PR axons are on the apical side of the CG membrane and are extended posteriorly through the optic stalk into the optic lobe.

The SGs constitute the major subtype of RBGs and have round shape morphology and molecular signatures of expressing *C527-GAL4*, Pdm3 and Seven-up (Svp)^14^. It migrates in the eye disc and is the only RBG subtype that can undergo cell division^14,18^. Of note, Svp is also expressed in CG^19^.

The WG exhibits elongated morphology and molecular signature of *Mz97-GAL4*, Cut, *sprouty-lacZ* (*sty-lacZ*) and *rau-lacZ* expression^14^. They do not divide^14,18,20,21^. *Mz97-GAL4* is a *GAL4* insertion in the first intron of the *öbek* gene, which encodes the N-terminal amidohydrolase 1^22^. Of note, Cut is also expressed in CG^23^. The WGs extend membrane to enwrap the photoreceptor axons and extend through the optic stalk and terminate in the optic lamina^24,25^.

The optic stalk is a single-cell layer tubular structure made of a distinct group of SG, characterized by *NP4702-GAL4* expression, and surrounded externally by basement membrane^24^. The SGs of OS are restricted to OS and do not extend into the eye disc^24^. Therefore, our analysis of SG in the eye disc does not involve the SG in the optic stalk.

The SGs are the precursor of WGs^14,18^. When cell division of SGs is blocked (*C527* > *fzr*), only 4–5 RBGs with large nucleus remained^14^, two of which are presumably the CGs. This result suggests that only 2–3 founder cells in an eye disc proliferated into all SGs and WGs. In contrast, blocking cell division in WG (*Mz97* > *fzr*), the RBG number is not affected, suggesting that the *Mz97*^+^ WGs do not divide. Clonal analysis also showed that WGs are derived from SGs^14,18^. It was reported that SG only lie at the basal-most surface, i.e. under the CG membrane, and WGs locate apical to the CG membrane^14,23^. It was suggested that because SG migrate under the CG membrane, they are prevented from contacting the PR axons, thereby can only receive a differentiation signal from the newly differentiated PR axons upon passing the anterior front of CG membrane, and then differentiate into WG^14^. In this “sequential differentiation model”, the CG membrane serves as a physical barrier to prevent the migrating SG from receiving the differentiating signal from PR axons^14^ (see below). Consistent with this model, our previous live imaging analysis found that new WG appear de novo at the anterior region (rather than migrating to the anterior), presumably differentiating from SG^18^.

The FGF signaling regulates multiple aspects of RBG, including SG proliferation and migration and WG differentiation. *Drosophila* has two FGFR receptors, namely Heartless (Htl) and Breathless (Btl), and three FGF ligands, namely Pyramus (Pyr), Thisbe (Ths) and Branchless (Bnl). These receptors and ligand have distinct expression patterns and affect different aspects off RBG behaviors. Bnl is expressed in a subset of R8 PR neurons in the eye disc^26^ and acts on Btl in SGs to prevent the precocious or overshot migration of SGs^27^. Pyr expressed in CG^15^ is important to promote SG proliferation and motility^28^. SG proliferation and migration also requires the POU domain transcription factor Pdm3^23^ and the N-terminal asparagine amidohydrolase homolog Öbek^22^. Ths is expressed in PR and is required to stop SG migration and to induce WG differentiation^28^. Both Ths and Pyr activate the FGFR Htl but act through different downstream signaling components to achieve distinct functions^23,28^. The MAPK Rolled and ETS domain transcription factor Pointed are FGFR downstream components required for WG differentiation, while the Rap1 is required for SG proliferation and migration^28^. Upon Htl signaling, the expression of the COUP transcription factor Seven-up (Svp) in SG is suppressed, thereby allowing WG differentiation^19^. In WG, Sty and Rau respectively suppress and promote FGFR signaling to maintains a critical level of FGFR signaling for the WG fate^28^. WGs then extend membrane to enwrap PR axons. The glia-axon recognition is dependent on the interaction of two transmembrane proteins, Borderless (Bdl) on WG and Turtle (Tutl) on PR axon^29,30^.

In this study, we examined the lineage relationship among CGs, SGs and WGs. A central point in the sequential differentiation model is that the SGs migrate under the CG membrane^13^, i.e. in the basal layer, thereby are prevented by the CG membrane from contacting the photoreceptor-derived Ths at the apical layer. Unexpectedly, we found that the SGs are present in both apical and basal layers. In this scenario, the apical SGs would not be kept from contacting the PR axons and CG may not be acting as a physical barrier. We found that the apical and basal SGs are derived from distinct lineages that become segregated before entering the eye disc. Furthermore, we found that only the apical SGs are competent to receive the FGF signal and differentiate into WG. We further used lineage tracing to provide a temporal profile of the developmental timing of the segregation of the different lineages.

## Result

### SGs are in both apical and basal layers

**Figure 1:**
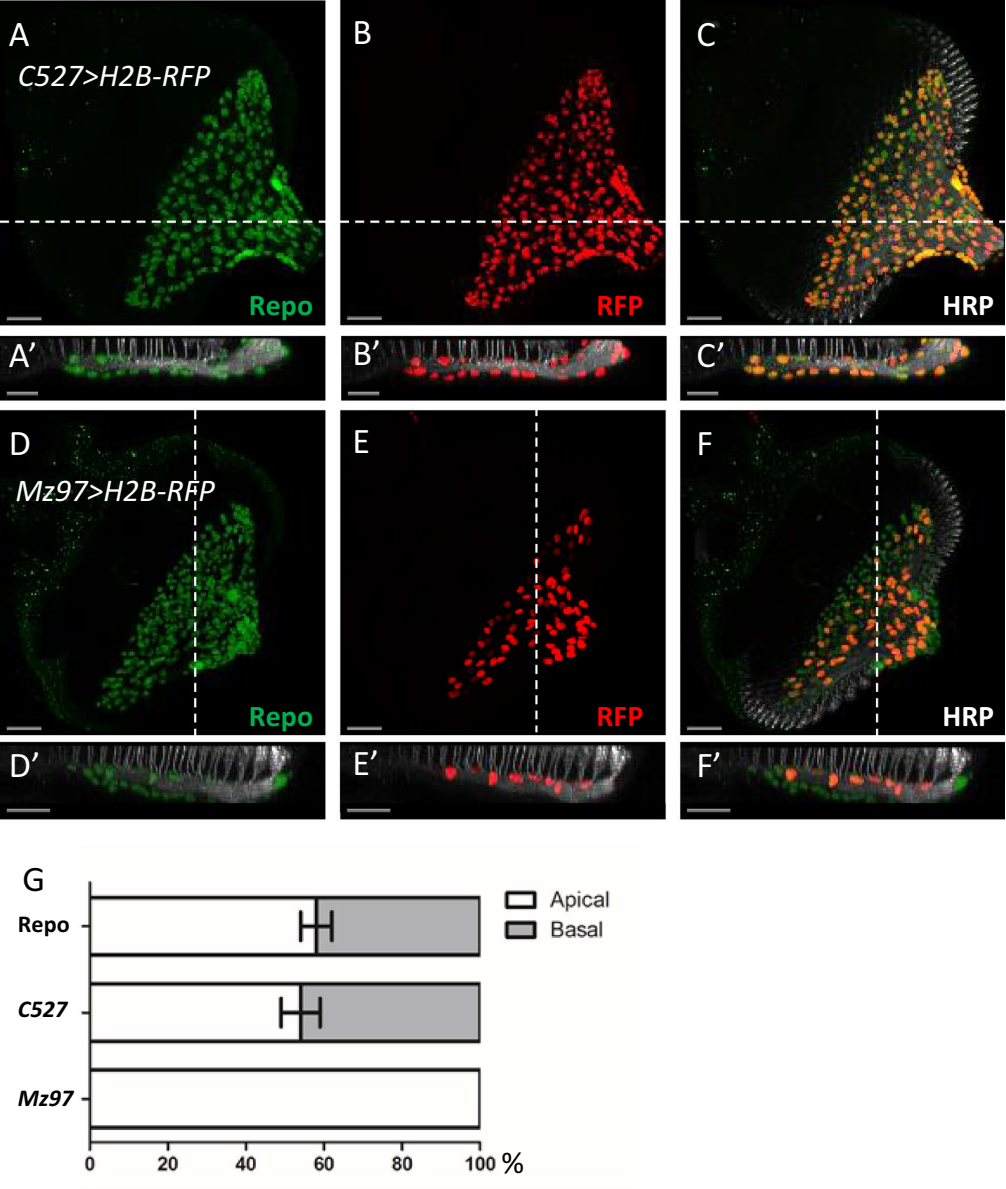
Apical-basal distribution of SG and WG. All panels represent late third instar larval eye discs. Anti-HRP antibody (white) labeled photoreceptor axons and served as a reference to distinguish the apical and basal layers of eye discs. (**A**–**C**) Eye discs with *C527-GAL4-*driven *H2B-RFP* (*C527* > *H2B-RFP*) were co-stained with anti-Repo and anti-HRP antibodies. (**A**) RBGs were labeled by anti-Repo antibody (green). (**B**) *C527* > *H2B-RFP* (RFP, red) marks the surface glia (SG). (**C**) The merge of RFP, Repo and HRP signals. (**A**′–**C**′) The lateral view along the dotted lines in (**A–C**), respectively. (**D–F**) *Mz97* > *H2B-RFP* eye discs were co-stained with anti-Repo and anti-HRP antibodies. (**D**) RBGs were labeled by anti-Repo antibody (green). (**E**) *Mz97* > *H2B-RFP* marks wrapping glia (WG, red). (**F**) The merge of the RFP, Repo and HRP signals. (**D**′–**F**′) The lateral view along the dotted line of (**D–F**). (**E**) Quantification of the relative apical and basal distribution of all RBGs, SGs and WGs in the eye disc. Percentage was calculated for each disc. Data are represented as mean ± S.D. n = 14 discs. Scale bars, 30 µm.

We first examined the apical-basal distribution of SGs in the eye disc. Along the apical–basal axis of the eye disc, the photoreceptors are apical to the CG membrane. The PR axon layer, marked by HRP, is apical to CG membrane. Accordingly, RBGs above or in the axon layer and RGBs below the axon layer were defined as apical and basal RGBs, respectively. We labeled RBG, SG, and WG nuclei, by anti-Repo antibody (Fig. 1A,D), *H2B-RFP* overexpressed by *C527-GAL4* (Fig. 1B; abbreviated as *C527* > *H2B-RFP*), *H2B-RFP* overexpressed by *Mz97-GAL4* (Fig. 1E; abbreviated as *Mz97* > *H2B-RFP*), respectively, and marked photoreceptor membrane by anti-HRP antibody (Fig. 1A′–F′). We found that 53% of RBGs and 50% of SGs are located in the apical layer (Fig. 1A′–F′), while 100% of WG are locate in the apical layer (Fig. 1G). We conclude that WGs are restricted to the apical layer, as previously reported^13^, but SGs are about equally distributed in both apical and basal layers, which is inconsistent with previous report^13^.

### Both apical and basal SGs can undergo cell division

**Figure 2:**
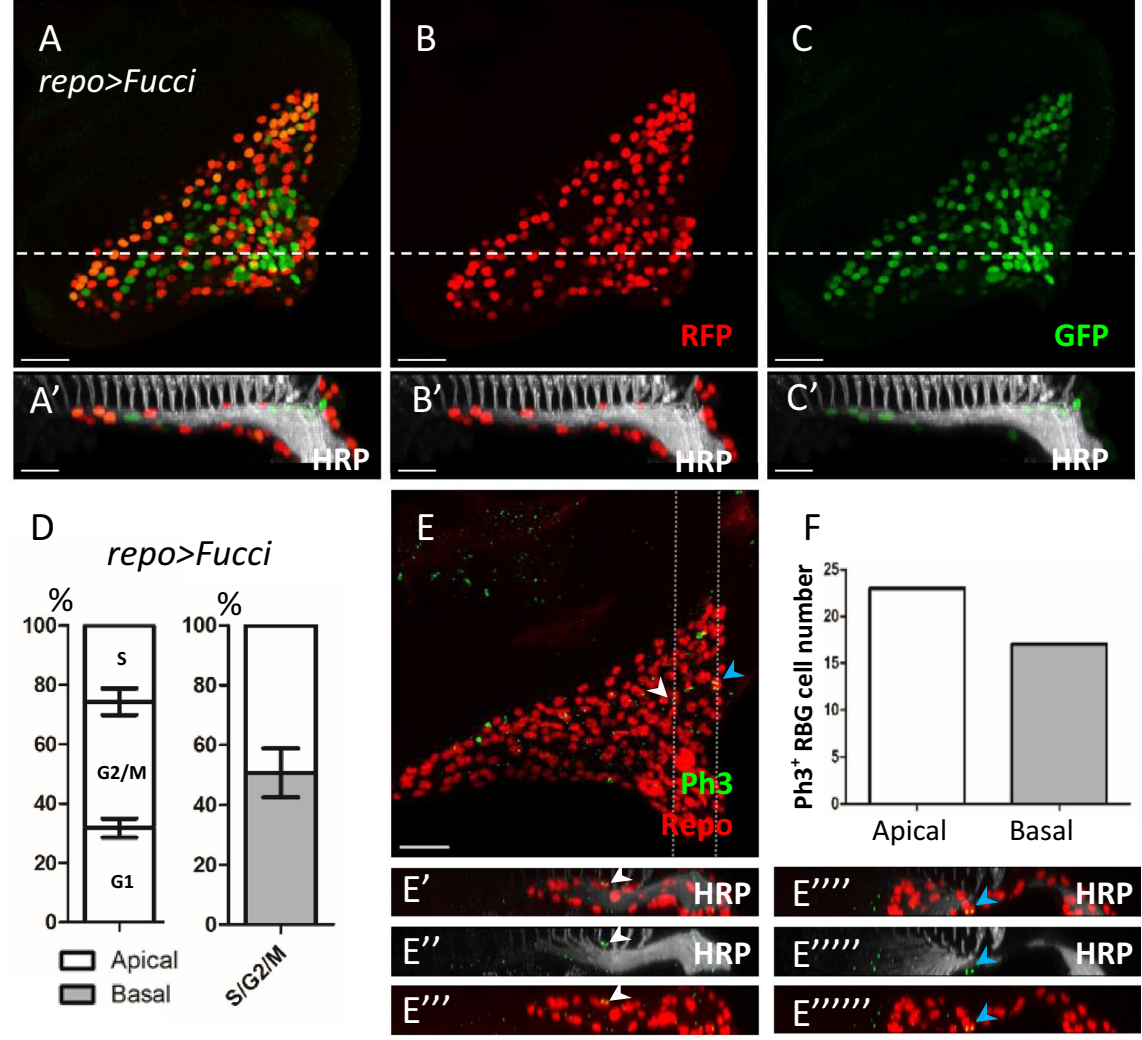
Both apical and basal SGs can divide. All panels represent late third instar larval eye discs. (**A–C**) *repo* > *Fucci* distinguishes the different cell cycle stages in all RBGs. (See text.) (**B**) The RFP (red) labels RBGs in S, G2, and M phases. (**C**) The GFP (green) labels RBGs in the G1, G2 and M phases. Their combinations allow unambiguous identifications of RGBs in G1 (GFP only; green), S (RFP only; red), and G2 (GFP and RFP; yellow) phases. (**A**′–**C**′) The lateral view along the dotted lines of (**A–C**). (**D**) Percentage of the RBGs in different cell cycle phases and the apical-basal distributions of RBGs in S/G2/M phase. The percentage is generated by dividing the cell number in different cell cycle stage by total RBG cell number. Data are represented as mean ± S.D., n = 12 discs. (**E**) The mitotic glia cells are labeled by anti-Repo (red) and anti-phospho-histone 3 (Ph3, green) antibodies. (**E**′–**E**′′′′′′) The lateral view along the left and right dotted lines of (**D**), respectively. The white and blue arrows indicate Ph3-positive RBGs. (**F**) Quantification of the apical-basal distributions of Ph3-positive RBGs in (**E**). Scale bars, 30 µm.

Since SGs are located equally on apical and basal layers, we examined whether SGs on both layers can undergo cell division. We used the Fucci (fluorescent ubiquitination-based cell cycle indicator) system^31^ to mark glia in different cell cycle phases. We expressed Fucci in all RBGs by *repo-Gal4* (Fig. 2A–C) and co-stained with anti-HRP antibody (Fig. 2A′–C′) to identify the cell cycle stage of RBG and their apical and basal distribution. 31.8 ± 3.1% of RBGs were in the G1 phase (labeled by GFP only), 25.7 ± 2.6% of RBGs were in S phase (labeled by RFP only), and 42.6 ± 4.4% of RBGs were in G2/M phase (labeled by both GFP and RFP) (Fig. 2D, left). Focusing on RBGs in S/G2/M phase, 50.7 ± 8.2% were located in the apical layer (Fig. 2D, right). Cell division was further examined by the mitotic marker phospho-histone H3 (Ph3) and co-stained with anti-Repo and anti-HRP antibodies (Fig. 2E–E′′′′′′). Collectively, 39 Ph3^+^ RBGs were observed in 12 eye discs and 22 of them were located in the apical layer (Fig. 2F). Since previous studies have shown that WGs are post-mitotic and all RBGs undergoing cell division are SGs^14,18^, our results suggest that both apical and basal SGs can divide.

### Identifying a transition stage in SG-to-WG differentiation

We found that 81% of RBG (Repo^+^) are co-labeled by the SG marker (*C527* > *H2B-RFP*), and 34% of RBGs are co-labeled by WG marker (*Mz97* > *H2B-RFP*). It suggests that there is 15% of RBGs that are *C527* and *Mz97* double positive. Since Cut is expressed in all WGs^23^, we checked its overlap with *C527*. We found that 22% of RBGs coexpressed *C527* > *nls-GFP* and Cut (Supplementary Fig. 1). Thus about 15–22% of RBGs are positive for both WG and SG markers and may represent an intermediate stage of the SG-to-WG transition. This is consistent with the competence stage suggested in a previous study^23^. Hereafter, we refer to cells at this transition stage as pre-WG (pWG). Since pWGs are *Mz97*^+^, so are expected to be post-mitotic. In contrast to the transitional pWGs, 11.7% of RBGs expressed only the WG marker Cut and negative for *C527*. This group likely represents the mature WG. These are located predominately in the posterior one-third section of the RBG population (Supplementary Fig. 1E). In contrast, the C527^+^ Cut^+^ pWG are predominantly located in the anterior one-third region (Supplementary Fig. 1F), consistent with the observation that new WG emerge at the anterior region^18^.

### SG-to-WG differentiation only occurs in the apical layer

**Figure 3:**
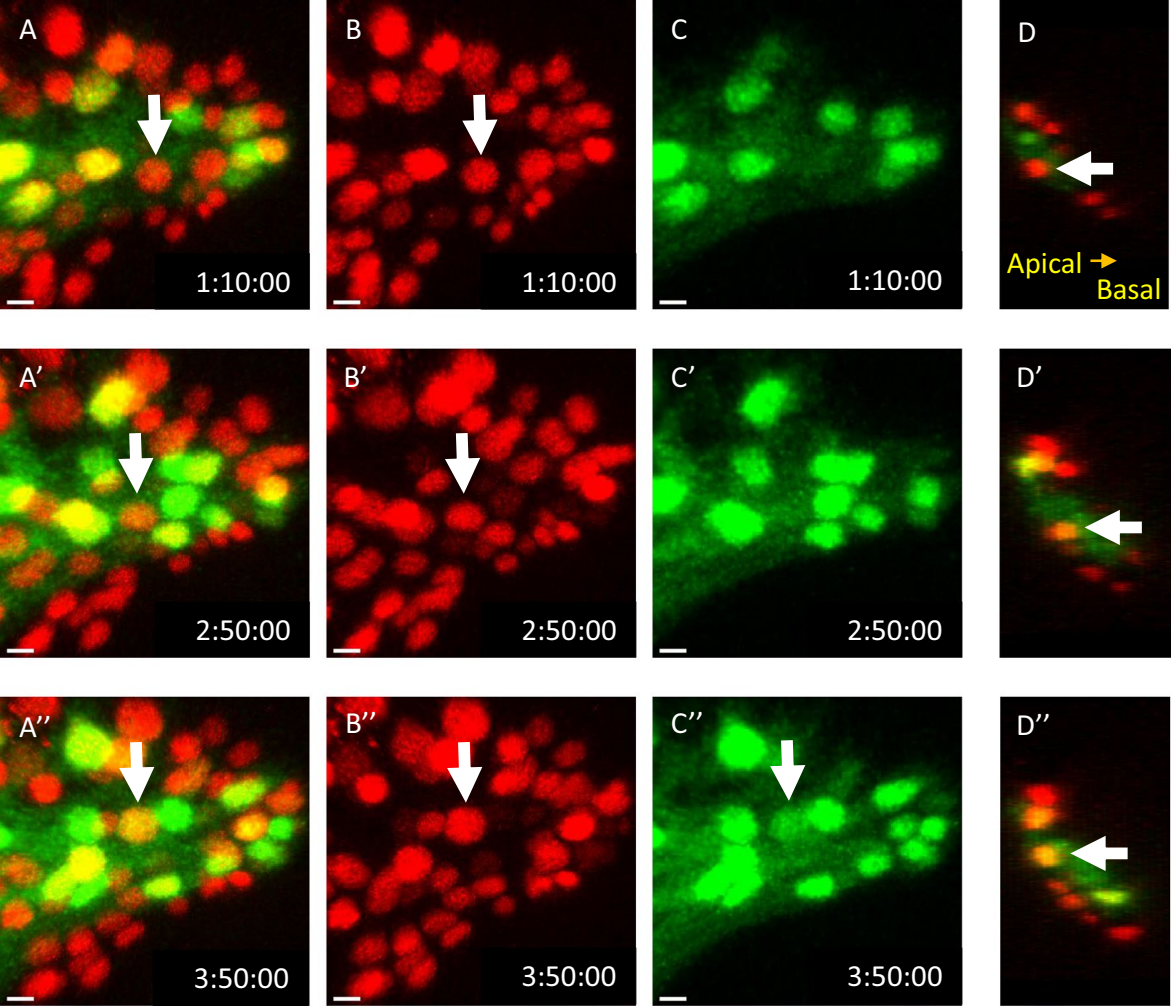
SG-to-WG differentiation only occur in the apical layer. Frames were taken from the S1. Movie of L3 eye disc cultured ex vivo at 25 °C for 10 h. RBG nuclei were labeled by repo-nRFP (red). WG nucleus was labeled by Mz97 > GFP (green). The arrow points out one glia cell differentiates from SG into WG. (**A–A**′′) The merged frame labeled the different subtype of RBG. The WG and SG were labeled by yellow and red, respectively. (**B–B**′′) The RBG was labeled by RFP. (**C**–**C**′′) The WG was labeled by GFP. (**D–D**′′) The lateral view of the frame. Three eye discs were each examined for ~ 10 h and 29.3 ± 2.6 (a total of 88) SG-to-WG events per disc were observed. Scale bars, 30 µm.

We next monitored SG-to-WG differentiation by live imaging. Because the SG and WG specific markers, *C527-GAL* and *Mz97-GAL4*, are both GAL4 drivers, this prevented us from using them together to directly follow SG-to-WG transition in live tissue. Instead, we labelled all RBGs by *repo-nlsRFP* (Fig. 3A,B, red) and labelled the WGs by *Mz97* > *GFP* (Fig. 3A,C, green). Since the *C527* > *nGFP* and *Cut* cells showed a complete overlap with Repo (Supplementary Fig. 1D), the *repo-nRFP*^+^, *Mz97*^*−*^ population is taken to represent the SG before the transition stage. During a 10 h live imaging of ex vivo cultured third instar eye disc (Supplementary Movie 1), the WG cell number increased from 43 to 76. We observed 33 *repo-nRFP*^+^ cells (about 10% of RBGs) turned from red to yellow (Fig. 3, arrow), indicating that the cell changed from expressing *repo* only (red) to expressing both *repo* and *Mz97-GAL4* (red + green). Before the SG-to-WG differentiation, these SG were located in the apical layer (Fig. 3D). Therefore, we captured the SG-to-WG differentiation events in action by live imaging, and confirm that these events occur only at the apical layer.

### Separation of apical and basal SG lineages

**Figure 4:**
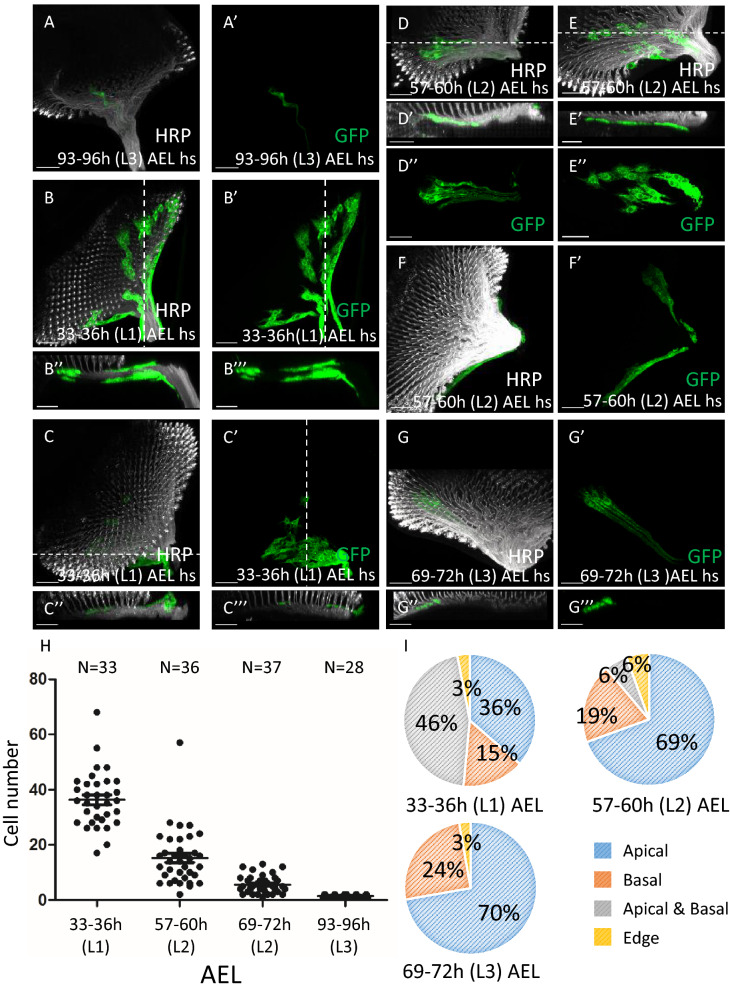
Early segregation of apical and basal RBG lineages. Flip-out clones were induced in the first (33–36 h AEL), second (57–60 h AEL) and third instar (69–72 h AEL) larva stage. Only RBG cells in a clone are visualized by *repo-GAL4*-driven *mCD8-GFP* (green). The HRP (white) serves as a reference to distinguish the apical or basal location of RBGs. (**A**, **A**′) The clone generated by a 10′ heat shock induction in 93–96 h after egg lay (AEL) were all single-cell clone and each disc has at most one clone. About 1000 eye discs were examined for each of the 33–36 h, 57–60 h and 69–72 h AEL time points, and about 800 eye discs were examined for the 93–96 h AEL group. (**B**, **C**) Clones generated at 33–36 h AEL. (**B–B**′′′) The cells in a clone occupied both the apical and basal layers. By morphology, these include both WG and SG. (**B**′′) and (**B**′′′) are lateral views from different sections [dotted line in (**B**) and (**B**′), respectively]. (**C**–**C**′) The cells in a clone are all located in the apical layer. By morphology, they are SG. (**C**′′) and (**C**′′′) are lateral views from different sections [dotted line in (**C**) and (**C**′′), respectively]. (**D–F**) Clones generated at 57–60 h AEL. (**D–D**′′) All cells in the single clone are in the apical layer. By morphology, these cells are WG. (**E–E**′′) All cells in the single clone are in the basal layer. By morphology, these cells are SG. (**D**′, **E**′) Lateral view. (**F**, **F**′) The cells in a clone are located at the eye disc margin. Their apical or basal location cannot be determined. (**G–G**′) Clones generated at 69–72 h AEL. All cells in a clone are in the apical layer. (**H**) The number of cells within single clones generated at different developmental time points. The number of discs examined are indicated. (**I**) Summary shows the percentage of apical and basal distributions of clones generated in L1, L2 and L3. Scale bars, 30 µm.

SGs are distributed in both apical and basal layers, but SG-to-WG differentiation occurs only in the apical layer. Do these apical SGs originate from the apical layer per se, or originate from the basal layer, migrate past the CG membrane and arrive at the apical layer before the SG-to-WG differentiation? Or can the basal SGs migrate through the CG membrane and onto the apical layer? We checked this by generating marked RBG clones early in development. If the apical SGs (aSG) are derived from the basal SG (bSG), then some of the cells in a clone may have reached the apical layer while the rest remain in the basal layer of the eye disc. Alternatively, if all cells within a clone are either all apical or all basal, the apical SGs should have an independent origin than that of basal SGs. Flp-out clones were generated at different early developmental stages and examined at late L3. The RBG clones were marked by *repo* > *mCD8-GFP*. We tested different duration of heat shock at 93–96 h after egg laying (AEL) to induce clone and examine at late L3. We found that a 10 min heat shock treatment induced at most a single one-cell clone in an eye disc (Fig. 4A–A′). Therefore, we used the 10 min heat shock for clone induction to ensure that the GFP^+^ cells in each disc are derived from a single parental cell. As expected, clones induced early can undergo more rounds of cell division so have higher cell numbers, whereas clones induced later have lower cell numbers (Fig. 4H). When clones were induced at L1 (33–36 h AEL), 46% of clones occupied both apical and basal layer (Fig. 4B,B′). This proportion dropped to 6% and 0% when clones were induced in L2 (57–60 h AEL) and in L3 (69–72 h AEL) (Fig. 4G,G′), respectively (Fig. 4I). In contrast, the proportion of clones that occupied only the apical layer increased from 36 to 69% and 70% when clones were induced at L1, L2 and L3, respectively (Fig. 4C,D,G,I). The proportion of clones that occupied only the basal layer increased from 15 to 19% and 24%, when clones were induced at L1, L2 and L3, respectively (Fig. 4E,I). Some clones are located at the edge and cannot be determined to be apical or basal (Fig. 4F). These results suggested that the apical and basal RBGs are derived from distinct lineages and that this lineage separation is nearly completed by the L2 stage. This timing is significant because the RBGs have not migrated into the eye disc at L2. Thus, the apical and basal lineage separation is a true lineage separation rather than simply due to their apical or basal location. The nuclear receptor Seven-up is expressed at high level in the apical SG and low level in the basal SG^19^. Therefore, the two lineages are molecularly different by at least one molecular marker.

### Only apical SGs are competent to respond to FGF signal for its differentiation into WG

**Figure 5:**
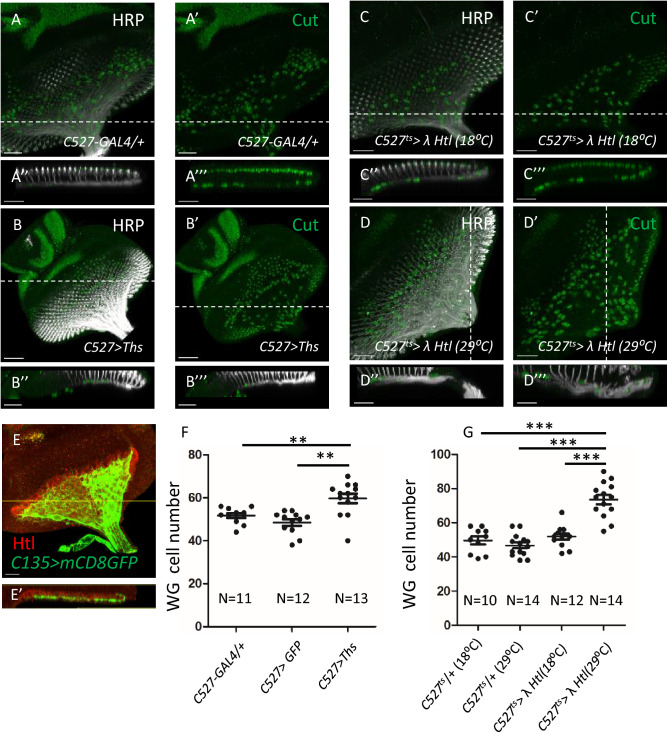
Only apical SGs are competent to respond to Ths-Htl signaling to differentiate into WG. All panels represent the eye disc in the third instar larva stage. HRP (white) was used to label the photoreceptor axon and serves as a reference to distinguish the apical and basal layers. Anti-Cut (green) was used to label the WG. (**A–A**′′′) C527-GAL4/+ as a control. (**B–B**′′′) Expression of *UAS-Ths* by the *C527-Gal4* driver (abbreviated as *C527* > *Ths*). (**C**–**C**′′′) *C527* > *λHtl* + *GAL80*^*ts*^ (abbreviated as *C527*^*ts*^ > *λ-Htl*) incubated in 18 °C. (**D–D**′′′) 0–3 h AEL *C527*^*ts*^ > *λ-Htl* embryos were collected, raised at 18 °C for 60 h and shifted to 29 °C for additional 36 h before dissection. (**A**′′, **A**′′′, **B**′′, **B**′′′, **C**′′, **C**′′′, **D**′′, **D**′′′) lateral view along the dotted line in (**A–D**). (**E**) The eye disc of *C135* > *mCD8GFP* (green) labeled the CG membrane was stained for Htl (red). (**E**′) The lateral view along the yellow line in (**E**). (**F**) Quantification of the effect on WG cell number by expressed Ths in SG. The number of discs examined are indicated. (**G**) Quantification of the effect on WG cell number by expressed λHtl in SG. The number of discs examined are indicated. Independent samples t-test was used to assess the mean deviation of each column. Mean ± S.E.M.; symbols: *NS* not significant; p > 0.05; *p < 0.05; **p < 0.01; ***p < 0.001. Scale bars, 30 µm.

It has been reported that the photoreceptors secret the FGF ligand Thisbe (Ths) to induce WG differentiation^19,28^. The sequential differentiation model proposes that the basal SGs are prevented from receiving Ths by the CG membrane. Therefore, we checked the competence of responding to Ths-Htl signaling in both apical and basal SGs. We first examined the Htl expression by the Htl antibody (Fig. 5E). The Htl expression was detected only above the CG membrane, which is marked by *C135* > *mCD8GFP* (Fig. 5E′), suggesting that the basal SGs do not express Htl. Next, we expressed Ths in all SGs (*C527* > *Ths*), thereby providing Ths to both apical and basal SGs (Fig. 5B). This caused a significant increase in the number of WGs labelled by anti-Cut antibody (Fig. 5B, compare with Fig. 5A for the control *C527-GAL4* alone; data summarized in Fig. 5F). All WGs are located in the apical layer (Fig. 5A′,B′), suggesting that only the apical SGs can respond to Ths and differentiate into WG. We then expressed λ-Htl, a constitutively active form of FGF receptor, in all SG (*C527* > *λ-Htl*). Because *C527* > *λ-Htl* caused larval lethality, we added *tub-GAL80*^*ts*^ (abbreviated as *C527*^*ts*^ > *λ-Htl*) and shifted temperature from the permissive 18 °C to the non-permissive 29 °C only at L3. This manipulation limited *C527-GAL4* function to only L3 stage, so as to bypass the early detrimental effects by λ-Htl. Again, the WG cell number increased significantly (Fig. 5D, compared with control in 5C; quantification in 5G) and all WGs were in the apical layer (Fig. 5C′,D′). These analyzed discs were at comparable developmental age by matching the number of ommatidia rows and the total number of RBGs (Supplementary Fig. 2). Pan-glial expression of λ-Htl caused an increase of the WG marker *sty-lacZ* only in the apical surface^28^. These results confirmed that only the apical SGs can respond to Ths-Htl signal to differentiate into WGs (based on Cut and *sty-lacZ* expressions). Since the basal SGs cannot differentiate into WGs even when provided with an activated Htl, they may lack a downstream signaling component or have an active block at a step downstream of the receptor Htl. Taken together, our results suggested that only the apical SGs are competent to respond to Ths-Htl signal and differentiate into WGs. These results further supported that the apical and basal SGs are distinct cell lineage with different differentiation capability.

### Origin of the CG and SG lineages

We used twin-spot MARCM^32^ to label the two mitotic daughter cells and follow their lineages. Clones were generated in different developmental stages and examined at late third instar larval stage. Mitotic clones were induced by *hs-flp* to generate two daughter cells, one carrying *UAS-CD8-GFP* and the other carrying *UAS-CD2-RFP*. These reporter genes are restricted to be only expressed in glia cells by using *repo-GAL4*. The heat-shock duration was 5 min so that each disc would have at most a single flp event.

**Figure 6:**
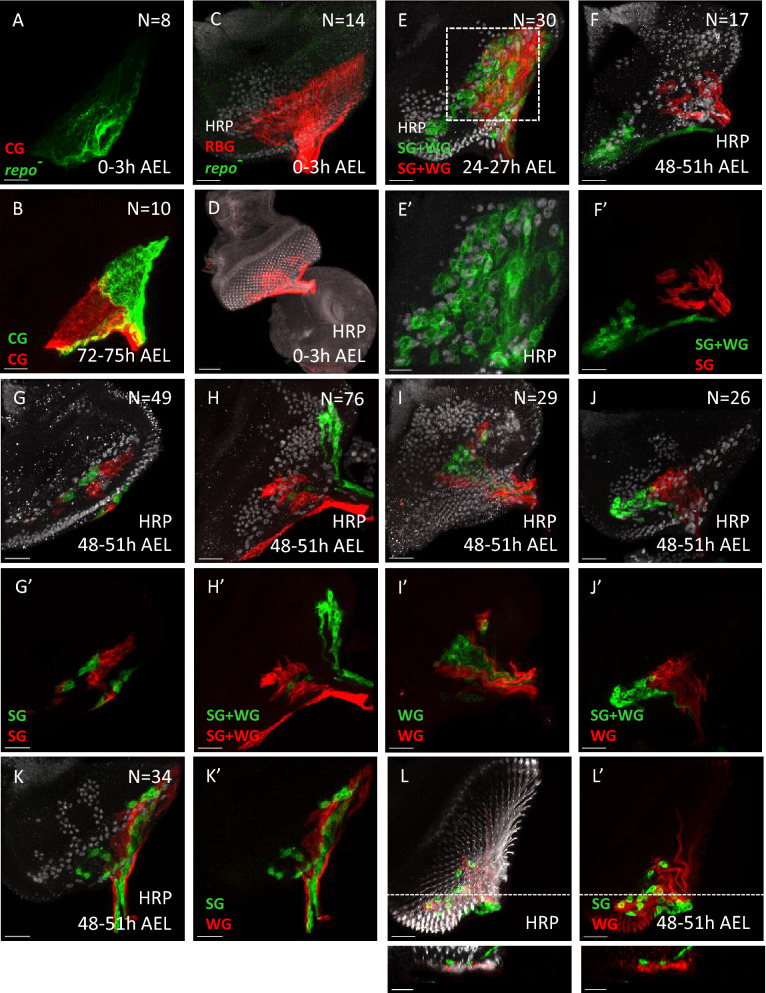
The RBG lineage analysis by twin-spot MARCM. Examples of tw-MARCM clones detected by *repo-GAL4*-driven reporters (CD8-GFP, green; CD2-RFP, red). Mitotic recombination was introduced by hs-FLP induced at different developmental stages. (**A**) Both CGs were labeled with a single reporter (GFP in this case) when clone was induced at 0–3 h AEL (n = 8). (**B**) Two CGs were respectively labeled with GFP and RFP when clone was induced at 72–75 h AEL (n = 10). (**C**, **D**) In clones generated at 0–3 h AEL (n = 14), only one sister clone was labeled, which was solely with non-CG RBGs, while the other sister clone was not detected in the eye disc, the optic stalk and the brain. (**C**) and (**D**) are different samples. (**E–J**) In clones generated at 24–27 h AEL (**E**) and 48–51 h AEL (**F–L**), SGs and WGs can be distinguished by their morphologies. WGs are also identified by anti-Cut antibody (white). (**E**) The two sister clones each contain SGs and WGs (n = 30). (**E**′) is the enlarged view of the dashed box region in (**E**). (**F**) One sister clone (red) contained only SGs, while the other sister clone (green) contained both SGs and WGs (n = 17). (**G**) Both sister clones contained only SGs (n = 49). (**H**) Both sister clones contained SGs and WGs (n = 76). (**I**) Both sister clones contained only WGs (n = 29). (**J**) One sister clone (green) contained both SGs and WGs, while the other (red) contained only WGs (n = 26). (**K**, **L**) One sister clone contained only SGs, while the other sister clone contained only WGs. (n = 34). (**L**, **L**′) Of note, all SGs in this clone were located at the apical layer (bottom panels). Scale bars, 30 µm.

**Figure 7:**
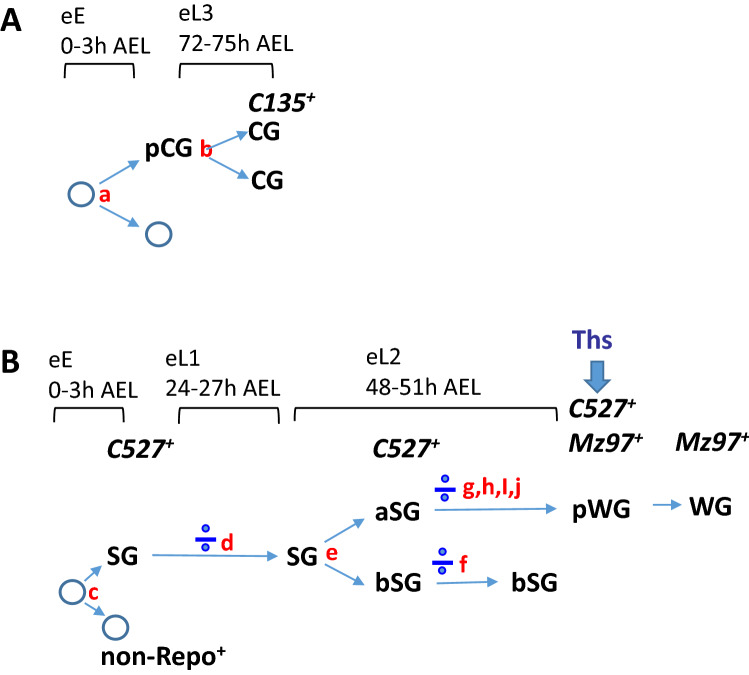
Temporal relationship of the specification of the RBG lineages. Our model for the consecutive generation of different RBG lineages. The time windows for clonal induction are indicated on the top. Proposed cell divisions that generate different clonal constitutions are marked in red alphabets. Symmetrical cell divisions are indicated by a mathematical division sign (÷). (**A**) The CG lineage. Division a (Fig. 6A): in early embryo (0–3 h AEL); generates one pre-CG (pCG) and one non-glia. Division b (Fig. 6B): in early L3 (72–75 h AEL); generates two CGs that begin to express *C135-GAL4*. Since CG are post-mitotic, the division is from one pCG (which does not express *C135-GAL4*) to generate two CGs. (**B**) The apical and basal SG and WG lineages. Division c (Fig. 6C): 2–3 founder cells exist in early embryo (0–3 h AEL). Each of these divides to generate one non-Repo^+^ cell and one non-CG RBG progenitor that begins to express *C527-GAL4* (SG). Division d (Fig. 6C): In L1 (24–27 h AEL), SG divides to generate more SG, some of which later differentiates into WG*.* Division e (Fig. 6F,F′,J): generates the apical and basal SG lineages. Both express *C527-GAL4*. This lineage segregation occurs before they migrate into the eye disc. The aSG migrate on top (apical) of CG membrane, while the bSG migrate below (basal) of CG membrane. Division f (Fig. 6E,E′): bSG divides to generate more bSG. There is no WG progeny. Division g (Fig. 6C): aSG divides to generate more aSG, some of which later becomes pWG with the coexpression of C527 and Mz97 and the competence to respond to Ths signaling and differentiate into WG. The differentiating WG loses C527 expression.

**Figure 8:**
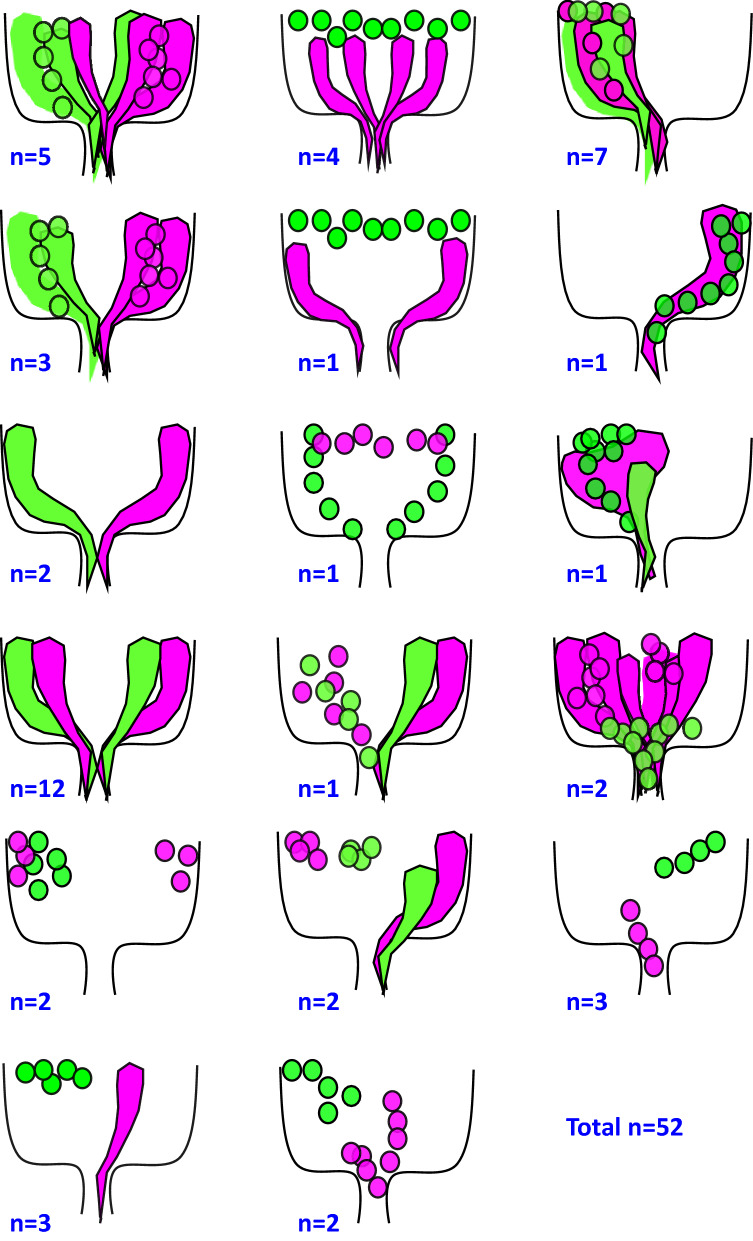
Differential spatial distributions of RBG cells within single clones and between sister clones. Graphic summary of the spatial distribution of cells derived from two sister clones generated by tw-MARCM. The two sister clones were marked magenta and green respectively. Cells are distinguished by morphologies: SG are rounded and WG are elongated. The number of cases for each type is indicated schematic diagram.

We first examined the CG lineage, which are recognized by membrane morphology. In about 800 eye discs subjected to clone induction at early embryo (0–3 h AEL), only eight discs have CG clones. In these, both CGs were labeled with the same reporter and no other RBGs were labelled (Figs. 6A, 8A, division a). This suggested that a division in the early embryo stage generated two daughter cells, one (the pre-CG) of which will divide to generate the two CGs at a later stage. This would explain why both CGs were labelled by the same lineage reporter. The other daughter cell derived from this early division is a non-RBG lineage, since no other RBGs were detected. In about 1200 discs with clone induction at early first instar (24–27 h AEL), no CG clone was detected. This suggests that the CG lineage did not undergo division during this period. In about 1000 discs with clone induction at early third instar (72–75 h AEL), 10 discs with CG clones were found. In these cases, the two CGs were marked by different reporter (Figs. 6B, 7A, division b). This set of results suggests that the pre-CG divided during 72–75 h AEL to generate the two CGs. Since our results suggest an early segregation of the CG and non-CG RBG fates, we further tested this idea by blocking cell division in the CG lineage. Blocking mitosis by expressing the cyclin-dependent kinase inhibitor Roughex (Rux) through the *C135-GAL4* (*C135* > *Rux*) did not affect the RBG cell number (Supplementary Fig. 3), supporting that the early segregation of the CG and non-CG RBG lineages. The observation of two CGs in these discs further suggested that *C135-GAL4* may not be expressed in the pre-CG (pCG). Its expression likely began after the division of pre-CG (Fig. 7A).

We next examined the non-CG RBGs. In about 800 eye discs with clone induction in early embryos (0–3 h AEL), 14 discs contained clones that each occupied only about 1/2 or 1/3 of the eye disc (Figs. 6C,D, 7B, division c). These results suggested that the founder population for the non-CG RBGs may consist of 2–3 precursor cells. Blocking mitosis by expressing Rux using *repo-GAL4* led to only 3–5 giant RBG cells in the eye disc (Supplementary Fig. 4B,B′). Expressing Rux by the SG-driver *C527-GAL4* gave similar result (Supplementary Fig. 4D,D′), while expressing of Rux by the WG-driver *Mz97-GAL4* did not affect the RBG cell numbers (Supplementary Fig. 4F,F′). These results (summarized in Supplementary Fig. 4G) are similar to those from a previous study^14^ with blocking mitosis by expressing Frz using *repo-GAL4*, *C527-GAL4* or *Mz97-GAL4*. These results suggest that 2–3 founder cells express *repo* and *C527-GAL4* and undergo cell divisions to generate the entire non-CG RBG population.

Interestingly, all cells in a given RBG clone induced in the early embryo (0–3 h AEL) were always of the same color, suggesting that their sister clones are not RBGs. We then checked whether their sister clones are glia that remain in the brain. However, no sister clone was found in brain and optic stalk (Fig. 6D). These results suggest that in early embryos, the cell division (Fig. 7B, division c) that generated the initial RBG precursor also generated a non-glia precursor, likely a neuroblast.

For the clones induced in the early first instar larvae (24–27 h AEL), both sister clones were detected in the eye disc (N = 30) (Figs. 6E, 7, division d). No disc with single-color clone was found. These data suggested that the non-CG RBG linage is already determined and can undergo cell division in the first larval stage. Within each clone, individual cell can be identified as SG and WG based on their morphology (Fig. 6E′). WGs were further identified based on Cut expression. Rounded cells with Cut expression were classified as WGs. According to the sequential differentiation model, one would expect that each clone would consist of SG cells that undergo cell divisions to generate more SGs that would progressively differentiate into WGs as they migrate anteriorly in the eye disc. The observation of WG-only and SG-only clones contradicts this model. Taking into account our findings that (a) anterior SGs and basal SGs are distinct lineages, (b) bSGs cannot differentiate into WGs, and (c) the existence of an intermediate stage pWG that expresses Mz97 and C527 and is non-dividing, the lineage relationships of different RBGs can be interpreted in a different model (Fig. 7).

Of the 231 discs with clones induced at early L2 stage (48–51 h AEL), eye discs can be categorized into six groups. Group 1 (17 clones) has SGs in one clone and SGs and WGs in the sister clone (Figs. 6F, 7B, division e), likely representing a division of a SG to generate an aSG and a bSG. Group 2 (49 clones) has both sister clones consisted only SGs (Figs. 6G, 7B, division f), likely representing a division of a bSG to generate two bSG. Group 3 (76 clones) has SGs and WGs within both sister clones (Figs. 6H, 7B, division g), likely representing an aSG dividing to generate two aSGs, which later further divide, with some progeny differentiate into WGs. Group 4 (29 clones) has both sister clones consisted only WGs (Figs. 6I, 7B, division h). Group 5 (26 clones) has WGs in one clone and SGs and WGs in the sister clone (in Figs. 6J, 7B, division i). Group 6 (34 clones) has SGs in one clone and WGs in the sister clone (Figs. 6K,L, 7B, division j). In group 6, the SGs are located at the apical layer (Fig. 6L). Groups 3–6 probably represents different stages of aSG-to-WG differentiation.

### Relationship among SG and WG in sister clones

We next used tw-MARCM to examine the relationship among the SGs and WGs derived from the two sister clones. 0–24 h-old embryos were collected and subjected to heat shock at 24–48 h AEL or 48–72 h AEL. The SGs and WGs were identified based on their morphologies. Consistent with the above findings, the sister clones can have different proportions of SGs and WGs. Interestingly, sister clones can have different cell numbers and different ratio of SGs and WGs. In one case, one clone has 12 GFP cells (4 SGs and 8WGs), while the sister clone has 37 RFP cells (7 SGs and 30WGs). This suggests that the sister clones may have different division rate. We cannot tell whether the difference in ratio of WGs and SGs is stochastic or inherent difference. The cells in the two sister clones may adopt different spatial distribution (Fig. 8), suggesting that the cells in the sister clones can migrate independently. Even cells within a clone can have different spatial distribution (anterior–posterior and dorsal–ventral) (Fig. 8). This further suggests that the migration of RBGs are independent of each other.

## Discussion

### Early segregation of SG into two distinct lineages with different differentiation capacity

Our findings showed that SGs are distributed equally in the apical and basal layer, relative to the CG membrane and PR axons. We found that the apical and basal SGs do not change their apicobasal position once they entered the eye disc, i.e. they cannot traverse the CG membrane. We found that the WGs only differentiates from apical SGs. This is not simply because the basal SGs do not have the opportunity to contact with the PR axons and receive the differentiation signal Ths. Instead, the basal SGs lack the Htl receptor. When we expressed Ths or constitutively activated Htl in all SGs, only the apical SGs responded to the Ths-Htl signal and differentiate into WGs; the basal SGs failed to do so. Cut is a marker for WGs and is required for WG differentiation^23^. Forced expression of Cut in SG caused the basal SGs to adopt WG-like morphology^23^. Cut does not induce full differentiation of WGs, since the WG marker *spy-lacZ* was not induced^23^. Therefore, the block of basal SGs to respond to Ths-Htl signaling is presumably at two levels, i.e. lack of the receptor Htl and a block at a step downstream of the receptor Htl but upstream of the nuclear effector Cut.

Is the difference of apical and basal SGs determined by their locations? Or is their difference in differentiation competence predetermined before they adopt their distinct apicobasal locations? Our lineage analysis showed that the two lineages become segregated by second instar larval stage, long before the RBGs enter the eye disc in third instar larvae. Therefore, their differences are determined not by the locations they adopt, but by a lineage decision.

Since the aSG is located, same as the PR axons, above the CG membrane, the CG membrane cannot serve as a physical barrier to prevent the migratory SG from receiving the differentiating signal from PR axons. Therefore, there must be a yet unidentified regulatory mechanism to provide the spatiotemporal coordination of SG-to-WG differentiation with the progressive differentiation of photoreceptor neurons.

### Lineage determines distinct migratory pathway

Since the apical and basal SGs derive from the same progenitors and segregate before they migrated into the eye disc, the decision to go under or above the CG membrane should be made before they encounter the CG membrane. That is to say, their lineage decision dictates them to choose distinctly migratory pathway. The apical and basal SG must have differential capacity to recognize guidance signals that can distinguish the apical and basal sides of CG membrane. It is possible that the two sides of CG membrane have different molecular signatures. Alternatively, the basal side has basement membrane, while the apical side has neurons. In vertebrates and invertebrates, glia can use integrin to interact with the extracellular matrix (ECM) in glial migration^33–35^. It is also possible that the difference is in photoreceptor axons, which lies only apical to the CG membrane. At the time of RBG migration into the eye disc, the first few rows of PRs have already extended their axons into optic stalk^14^. It is possible that the PR axons may send some signal(s) to attract the apical SGs at this early stage.

### Defining a transition stage in WG differentiation

We identified a population of RBGs that expresses markers for both WGs and SGs. This C527^+^, Mz97^+^ Cut^+^ population constitutes about two-thirds of WGs and about 22% of apical SG. We propose this apical population represents a transition state (pWG) from SG to WG (Supplementary Fig. 1). The C527^+^ Mz97^−^ apical SGs first become C527^+^ Mz97^+^ Cut^+^ and then turn into C527^−^ Mz97^+^ Cut^+^, which becomes differentiating WGs. Since Cut expression depends on FGFR signal^23^, the C527^+^ Mz97^−^ apical SG cells must have Htl and can respond to Ths to express Cut. *C527*^ts^ > *λ-Htl* caused an increase in WG number without affecting the total RBG cell number (data not shown). If the C527^+^ Mz97^−^ apical SG can respond to the activated FGFR and differentiate into the non-dividing WGs, the total RBG cell number should decrease, which is not the case of our results. Therefore, the C527^+^ Mz97^−^ apical SG is not competent to respond to FGFR signal. The C527^+^ Mz97^+^ pWG is the only stage that is competent to receive the FGFR signal for WG differentiation. The difference in competence is at a step downstream of the receptor Htl. In summary, the transition state pWG is defined both molecularly and functionally.

Live imaging showed that WG differentiation undergoes a series of morphological changes, going from round shape to progressive membrane elongation to enwrap axons and extend into the optic stalk^25^. It is not known how these morphological states correlate with the molecular states.

### Fate of the SG

The apical SGs have a chance to become WGs. It is not clear whether all apical SGs eventually become WGs in the end of eye disc development. If not, what happens to the remaining apical SGs? The basal SGs do not have the competence to become WGs and do not have a chance to see the FGF ligand Ths. What happens to the basal SGs later in development? It has been proposed that the SGs may corresponds to the fenestrated glia in the adult optic lamina^36^. However, the optic stalk SG expresses *NP-4702-GAL4*, but the eye disc SG does not^24^, suggesting that these are two distinct SG populations. Therefore, the eye disc SG are unlikely to extend into the optic lobe and thus cannot become the fenestrated glia in adult optic lamina. The fly blood–brain-barrier (BBB) is formed by perineurial glia and subperineurial glia^37^. The eye disc basal SGs may form, together with the CGs, the adult brain–retina barrier (BRB)^38^, and may play a role to maintain neuronal survival by providing important metabolites and in relaying body metabolic state to neural stem cells to control their exit from quiescence^39–41^.

### The glia-to-glia transition

Our results showed that one type of glia can differentiate into another type of glia. Part of this regulation is dependent on lineage, such that one lineage is competent for this transition and another lineage lacks this competence. Part of this transition depends on the differentiation signal from the photoreceptor neurons. This glia-to-glia transition may be a novel type of glia differentiation and may also exist in other systems.

In each developing leg disc, there are about 220 migratory perineurial glia (only a few are marked by *C527-GAL4*, while all are marked by anti-Apontic antibody), one WG and two subperineurial glia^42^. Therefore, there does not seem to be a progressive differentiation of perineurial (SG) into WG, as observed in the eye disc.

In the embryonic PNS, each larval abdominal segment has a fixed set of 12 glia, including three wrapping glial cells, four subperineurial glial cells and three perineurial glial cells. These are generated directly from neuroblasts (NB) or sensory organ precursors (SOP)^43,44^. Only one perineurial glia can undergo mitosis. The WG and subperineurial glia share the same progenitors^43,44^. Therefore, the generation of glia subtypes in the embryonic abdominal PNS does not seem to undergo a glia-to-glia transition.

In the embryonic CNS, the midline glia (MG) can be divided into anterior MGs (AMG) and posterior MGs (PMG) that differ in their positions, migration, gene expressions and functions^45–47^. However, whether these AMG and PMG come from the same progenitor is not clear.

In vertebrates, in vivo lineage tracing of single genetically marked neural progenitor cells (NPC) showed that some NPCs generate both neurons and glia, while other NPCs generate only specific glia types (astrocytes or oligodendrocytes) or only neurons^48^. There is no example that one type of glia derives from another type of glia.

Lineage tracing of the astrocytes in the olfactory bulb showed that the progeny cells of an astrocyte clone can adopt different morphologies, which is dependent on its position within the olfactory bulb^49^. This is similar to the case in the fly eye disc, where lineage differences coupled with spatial signal provides the final differentiation outcome.

## Material and methods

### Fly stocks

The fly stocks used are *UAS-H2B-RFP* (Langevin et al., 2005), *repo-RFP.nls*^50^, *UAS-Fucci*^31^, *C135-Gal4*^15^, *elav-Gal4*, *C527-Gal4*, *MZ97-Gal4*, *UAS-ths*, *UAS-λ-htl*, and *UAS-GFP.nls* were from Bloomington Drosophila Stock Center. Twin-spot MARCM tool was a gift from Dr. Hung-Hsiang Yu.

### Live imaging of eye disc

Larval discs were dissected and imaged followed by the protocol as previously described^18^. Schneider’s Drosophila medium (Thermo 21720-024) was supplemented with 2% FBS (Sigma N4765), 0.5% penicillin–streptomycin (GIBCO0759), and 1.25 mg/ml insulin (Sigma I9278). Prepared culture medium should be used within a month. 0.75% low-gelling-temperature agarose (Agarose II, AMRESCO 0815-25G) in 1xPBS was added to hold the tissue for imaging. The ex vivo time-lapse images were acquired with Zeiss LSM 710 with a GaAsp detector equipped with a culture chamber maintained at 25 °C and continuous support of humid air (25 °C). C-Apochromat 40×/1.2 W Korr objectives were used for the imaging. Z-stack images were acquired every 5 min as indicated in the movies. 30–60 slices per stack were used based on the type of fluorescent proteins (nucleus/membrane) from different samples. Optical sections were set to 1.2 µm with an optical interval of 1 µm. Time is displayed as h:min:s, relative to the start of the time-lapse.

### Heat shock for clone induction

The eggs were collected for 3 h. The heat shock was conducted for in 37 °C water bath and dissected at 120 h after egg lay. The heat shock duration was 10 min for flp-out clone. The heat shock duration was 5 min for twin spot MARCM.

### Immunostaining

Larval discs were dissected, fixed and stained followed by the protocol as previously described^51^. Primary antibodies were used: mouse anti-Cut (1:100) and mouse anti-Repo (1:100) from Developmental Studies Hybridoma Bank (DSHB, University of Iowa), Rabbit anti-Phospho H3 (1:500) from Millipore. Goat anti-Htl was a gift from Dr. Christian Klämbt. Fluorescence conjugated secondary antibodies, including anti-HRP, were obtained from Jackson ImmunoResearch. Imaging procedures were acquired by LSM 880 confocal microscope (Zeiss).

## Supplementary information

Supplementary Legends.

Supplementary Figures.

Supplementary Video 1.

Supplementary Video 2.
