# Supplementary material for: Early lineage segregation of the retinal basal glia in the Drosophila eye disc
Source: Sci Rep. 2020 Oct 28;10:18522. doi: 10.1038/s41598-020-75581-w (PMC7595039; doi:10.1038/s41598-020-75581-w)
Supplement: Supplementary file 1 — Supplementary Legends. [file 41598_2020_75581_MOESM1_ESM.pdf]

Early lineage segregation of the retinal basal glia in the *Drosophila* eye disc

Chia-Kang Tsao[1,2], Yu Fen Huang[1,2,#], and Y. Henry Sun[1,2,*]

Supplementary Fig. 1

(A-D) Eye discs with SGs labeled by *C527>nGFP* (green) were co-stained for Cut (red) and Repo (white). (E) Percentage of cells with *C527* and/or Cut in the Repo+ population. (F,G) The region of RBG in eye disc is divided into three sections (anterior, middle, posterior). (F) The percentage of C527^{-} Cut+ cells (representing more mature WG) within the three sections clearly showed that the more mature WGs are distributed predominantly in the more posterior section. (G) The percentage of C527+ Cut+ cells (representing the transition state) within three sections clearly showed that the cells at transition state are predominantly in the anterior one-third region. The Cut^{+} cells in the anterior of MF are hemocytes.

Supplementary Fig. 2 Control for the developmental stage of eye discs when expressing λHtl

For the experiment *C527^{ts}>λ-Htl* at 18°C (A, A') and 29°C (B, B') and for *C527^{ts}>Ths* at 18°C and 29°C described in Fig. 5, we controlled for the maturity of the eye disc for their number of ommatidia rows and number of total RBG cells.

Supplementary Fig. 3 The *C135-GAL4*-exprressing CGs are non-dividing

(A, B) In *C135>mCD8GFP*, the CG membrane extension is visualized by mCD8GFP (green). The RBGs are detected by anti-Repo (red). (C, D) In *C135>mCD8GFP+Rux*, the CG membrane (mCD8GFP, green) and the number of RBGs (Repo, red) are not affected. (E) The number of Repo^{+} cells in B and D are quantified. There is no significant difference.

Supplementary Fig. 4 The effect of blocking mitosis in different RBG populations

(A) *repo>nlsGFP*. (B, B') *repo>nlsGFP+Rux*. (C) *C527>nlsGFP*. (D, D') *C527>nlsGFP+Rux*. (E) *Mz97>nlsGFP*. (F) *Mz97>nlsGFP+Rux*. The RBG are marked by Repo (red). Neurons are marked by HRP (blue). GFP (green). (G) The number of Repo+ cells in the eye disc in (A-E) are summarized. Scale bars, 30 um.

Supplementary Movie 1 Live imaging of SG-to-WG transition in ex vivo cultured eye disc

The movie is taken from a L3 eye disc cultured *ex vivo* at 25°C for 10 hr. RBG nucleus was labeled by *repo-nRFP* (red). WG nucleus was labeled by *Mz97>GFP* (green). The arrow points out one glia cell differentiates from SG into WG.

Supplementary Movie 2 Live imaging of RBG migration among CG membrane in ex vivo cultured eye disc

The movie is taken from a L3 eye disc cultured *ex vivo* at 25°C for 7.5 hr. RBG nucleus was labeled by *repo-nRFP* (red). Carpet glia membrane was labeled by C135>moesinGFP (green).
