# Supplementary material for: Early lineage segregation of the retinal basal glia in the Drosophila eye disc
Source: Sci Rep. 2020 Oct 28;10:18522. doi: 10.1038/s41598-020-75581-w (PMC7595039; doi:10.1038/s41598-020-75581-w)

Early lineage segregation of the retinal basal glia in the *Drosophila* eye disc

Chia-Kang Tsao[1,2], Yu Fen Huang[1,2,#], and Y. Henry Sun[1,2,*]

Supplementary Fig.1

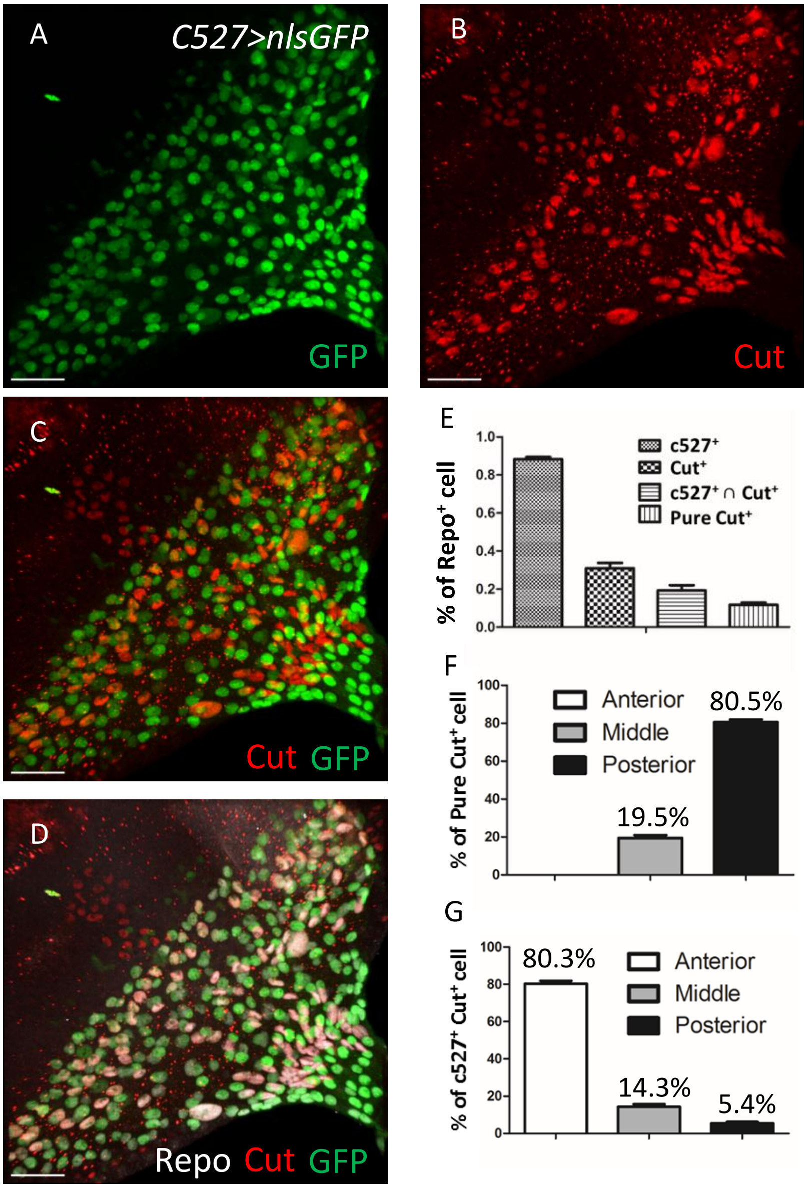

Supplementary Fig.2

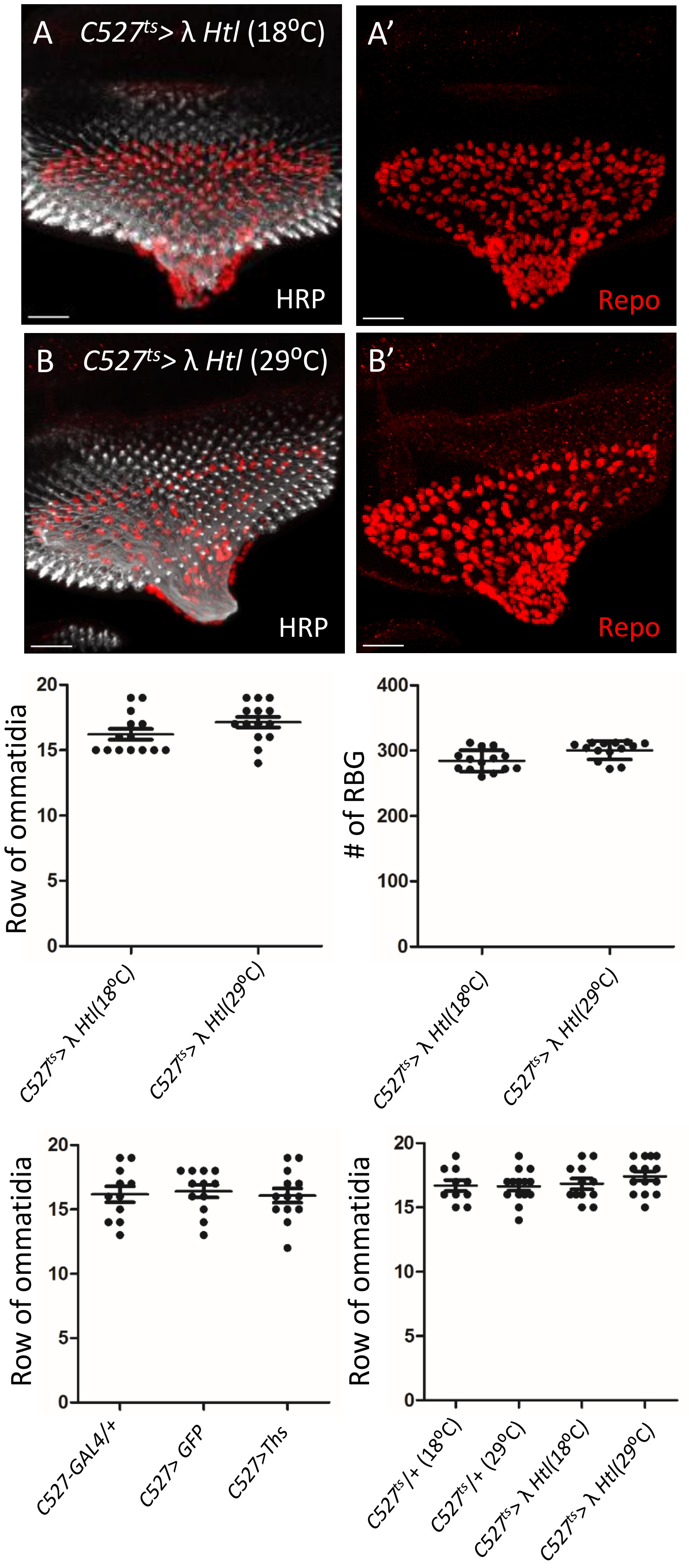

Supplementary Fig.3

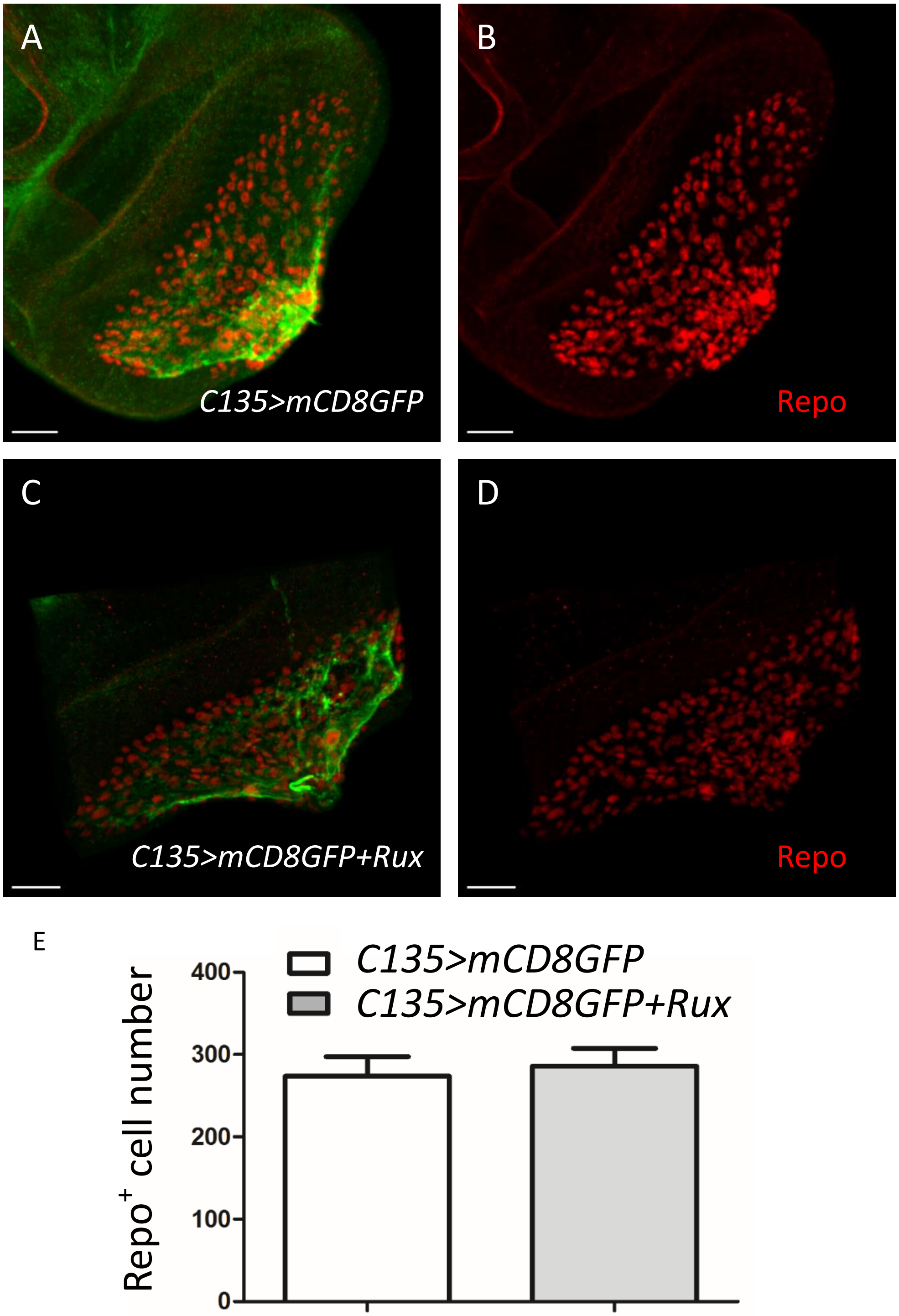

Supplementary Fig.4

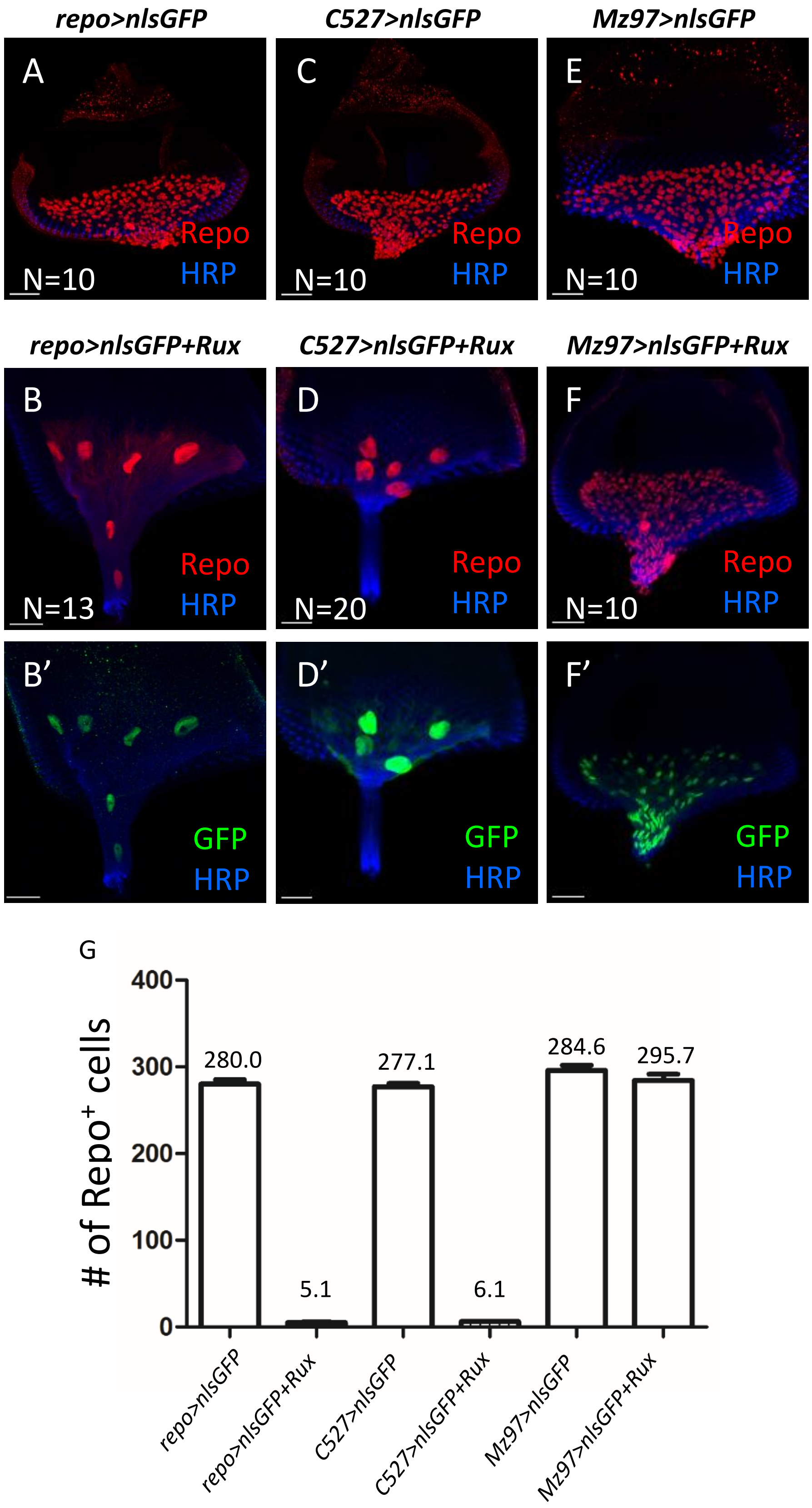

Supplement: Supplementary file 2 — Supplementary Figures. [file 41598_2020_75581_MOESM2_ESM.pdf]
